# Rapid Spread and Diversification of Respiratory Syncytial Virus Genotype ON1, Kenya

**DOI:** 10.3201/eid2006.131438

**Published:** 2014-06

**Authors:** Charles N. Agoti, James R. Otieno, Caroline W. Gitahi, Patricia A. Cane, D. James Nokes

**Affiliations:** Kenya Medical Research Institute–Wellcome Trust Research Programme, Kilifi, Kenya (C.N. Agoti, J.R. Otieno, C.W. Gitahi, D.J. Nokes);; Public Health England, London, UK (P.A. Cane);; University of Warwick and WIDER, Coventry, UK (D.J. Nokes)

**Keywords:** attachment protein, G glycoprotein, G protein gene, phylogenetic analysis, ON1, genotype ON1, respiratory syncytial virus, RSV, viruses, respiratory viruses, pneumonia, epidemics, humans, surveillance, Kenya

## Abstract

Respiratory syncytial virus genotype ON1, which is characterized by a 72-nt duplication in the attachment protein gene, has been detected in >10 countries since first identified in Ontario, Canada, in 2010. We describe 2 waves of genotype ON1 infections among children admitted to a rural hospital in Kenya during 2012. Phylogenetic analysis of attachment protein gene sequences showed multiple introductions of genotype ON1; variants distinct from the original Canadian viruses predominated in both infection waves. The genotype ON1 dominated over the other group A genotypes during the second wave, and some first wave ON1 variants reappeared in the second wave. An analysis of global genotype ON1 sequences determined that this genotype has become considerably diversified and has acquired signature coding mutations within immunogenic regions, and its most recent common ancestor dates to ≈2008–2009. Surveillance of genotype ON1 contributes to an understanding of the mechanisms of rapid emergence of respiratory viruses.

## Abstract 2

Surveillance of this new genotype helps clarify the mechanisms of rapid emergence of respiratory viruses.

Human respiratory syncytial virus (RSV) is the major viral cause of bronchiolitis and pneumonia in infants and also a major cause of severe respiratory illness in the elderly (*1*). RSV infection usually occurs in annual epidemics, and the virus can re-infect persons throughout life. RSV isolates fall into 2 groups, A and B, and each group includes multiple genotypes. RSV epidemics are often caused by several variants of >1 RSV genotypes, and the dominant genotype is usually replaced each year (*2*). RSV’s most variable protein, the attachment (G) glycoprotein, is also a target of protective antibody responses, and analysis of its encoding genome portion shows continuous accumulation of genetic changes leading to antigenic drift (*3*,*4*). However, as a nonsegmented, single-stranded RNA virus, RSV does not show the abrupt antigenic changes that are sometimes seen in influenza A viruses. The abrupt changes in influenza A viruses commonly arise when genome segments reassort, sometimes acquiring new surface protein genes from animal sources, leading to antigenic shift as was seen in the recent influenza A(H1N1) pandemic strain (*5*). Nevertheless, twice in recent years, a distinct new genotype of RSV has arisen as a result of duplication within the G gene. The first of these new genotypes was detected in 1999 when 3 group B viruses with a 60-nt duplication in the C-terminal region of the G gene, which encodes strain-specific epitopes (*4*), were isolated in Buenos Aires, Argentina (*6*). This genotype was also observed in a retrospective analysis of RSV samples from 1998 to 1999 in Madrid, Spain (*7*). This novel genotype spread rapidly and by 2003 was being detected around the world; by 2006, it had become the predominant group B genotype (*7*,*8*).

In December 2010, a novel RSV group A genotype, ON1, with a 72-nt duplication in the C-terminal region of the G gene, was detected in Ontario, Canada (*9*). This genotype was also detected in Malaysia, India, and South Korea at the end of 2011 (*10*–*12*) and in Germany, Italy, South Africa, Japan, China, and Kenya in 2012 (*13*–*15*) (GenBank, unpub. data). The emergence and spread of these new genotypes, which can be readily tracked by G gene sequencing, provide an opportunity to re-examine 1) the interconnectedness of RSV epidemics at various levels (e.g., global, country, and community levels), 2) the spatial–temporal scale of the spread of variants, and 3) the pace and nature of associated genetic changes. Such examinations have the potential to bring new insights regarding how RSV persists to cause recurrent epidemics in human populations.

We conducted a detailed analysis of G gene variability of the ON1 genotype viruses detected among children inpatients at a hospital in rural Kenya in 2012. Two RSV epidemics were observed during the year, and a wave of genotype ON1 cases occurred in each. We compare the phylogenetic relationship between the ON1 viruses detected in Kenya and ON1 viruses worldwide during a similar period.

## Materials and Methods

### Study Location and Participants

The study specimens were obtained from children <5 years of age who had been admitted with severe pneumonia to Kilifi District Hospital (KDH), Kenya, during 2012. All children were enrolled as part of an ongoing study, initiated in 2002, of the epidemiology and disease of RSV-associated pneumonia in case-patients (*16*–*18*). KDH, located in the coastal town of Kilifi, north of Mombasa, serves a rural (predominantly) and semiurban community. In this setting, epidemics of RSV disease occur on an annual basis, beginning in late October or early November of each year and continuing through June, July, or August of the next year (*18*).

### Clinical Samples and Laboratory Methods

Since 2002, nasal wash or nasopharyngeal swab specimens have been collected from all children enrolled in the pneumonia study. The samples are tested for the presence of RSV antigen and/or nucleic acid by using the indirect fluorescence antibody test and real-time reverse transcription PCR, respectively (*19*). G gene sequencing is routinely undertaken on all samples from the KDH study site that have RSV-positive test results (*16*,*20*) (J.R. Otieno and colleagues, unpub. data). The ON1 genotype was first detected by this surveillance in February 2012. This report focuses on RSV A specimens collected during 2012. 

Viral RNA extraction, reverse transcription, PCR amplification, and sequencing of the G gene were undertaken as described (*16*,*20*,*21*). The specimens were collected after informed consent was given by a parent or guardian for each child. The Kenya National Ethics Review body approved the study protocols.

### Sequence Alignments and Comparison Dataset

Consensus G gene sequences for RSV A were initially aligned in MAFFT v6.884b (*22*) and trimmed in BioEdit (http://www.mbio.ncsu.edu/bioedit/bioedit.html). Viruses possessing the ON1 72-nt duplication were readily identifiable from the alignments. Sequences for Kilifi viruses corresponded to the terminal 702 nt and 630 nt in the G ectodomain regions of ON1 and non-ON1 viruses, respectively. A comparison dataset of ON1 genotype sequences deposited into GenBank was downloaded, collated, aligned with the Kilifi ON1 sequences, and used to derive a global phylogenetic tree. Because some sequences were limited in length, the final worldwide ON1 alignment was trimmed to include only the C-terminal G gene region over the terminal 333 nt.

### Phylogenetic Analysis

Phylogenetic trees were constructed in MEGA5.2.1 (*23*) by using the maximum likelihood method under the general time-reversible model of evolution. The robustness of the phylogenetic clusters was evaluated by bootstrapping with 1,000 iterations. Viruses were considered to be the same variant if they were identical in nucleotide sequence over the region we sequenced. Variants were grouped into a similar lineage if they shared signature-coding mutations. The Kilifi sequences reported here are deposited in GenBank under accession numbers KF587911–KF588014.

### Evolutionary Analysis

The rate of evolution of the ON1 G gene and the date of the most recent common ancestor (MRCA) of the viruses collected to date were estimated by 2 independent methods: 1) by using regression of the root-to-tip distances from the maximum-likelihood tree in Path-O-gen (http://tree.bio.ed.ac.uk/software/pathogen/) and 2) by using the BEAST v1.74 analysis package (https://code.google.com/p/beast-mcmc/), which uses the Bayesian Markov chain Monte Carlo approach (*24*). The analysis included only sequences for which the exact date of sampling was provided. Furthermore, to reduce the bias of oversampling from any 1 location, we included only viruses with unique nucleotide sequences in the C-terminus region in the analysis. The final data subset comprised 65 sequences from 7 countries.

The BEAST analysis was run through 50 million steps, with sampling every 2,500 steps, under the HKY model of evolution and the Bayesian skyride population growth model. Once the analysis was complete, run convergence was confirmed by using the Tracer v1.5 program (http://tree.bio.ed.ac.uk/software/tracer/); trees were summarized in TreeAnnotator and visualized in FigTree v1.40 (http://tree.bio.ed.ac.uk/software/figtree/).

## Results

**Figure 1:**
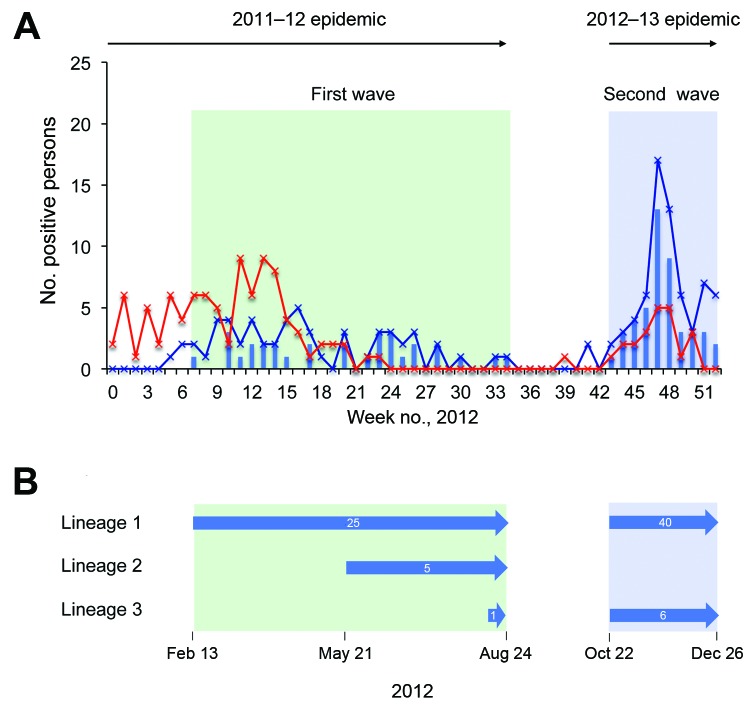
A) Number of persons positive for respiratory syncytial virus (RSV) genotype ON1 during 2 infection waves, Kilifi, Kenya, 2012. Blue line indicates cases of RSV group A infection; red line indicates cases of RSV group B infection; bars indicate number of ON1 case-patients admitted to Kilifi District Hospital during January 1–December 31, 2012. The first ON1 infection wave (green shading) overlapped with a 2011–12 RSV epidemic, and the second ON1 infection wave (blue shading) overlapped with a 2012–13 RSV epidemic (see arrows above graph). B) Lifespans of the main ON1 lineages observed at Kilifi. Numbers inside arrows indicate the number of sequences for the lineage named at the far left.

During January 1–December 31, 2012, a total of 873 children who were admitted to KDH were eligible for the RSV surveillance study. Nasal wash or nasopharyngeal swab specimens were obtained from 834 of the children and tested for RSV. Of the 834 samples, 240 (28.8%) were RSV positive: 123 (51.3%) were group A infections, 114 (47.5%) were group B infections, and 3 (1.3%) were A/B co-infections. Of the 126 combined group A and group A/B viruses, 104 (82.5%) were successfully sequenced in the G gene ectodomain region, and of these, 77 (74.0%) possessed the ON1 genotype 72-nt duplication. The numbers of RSV A, B, and ON1 cases detected each month at KDH during 2012 are shown in Table 1. The number of cases detected each week is shown in Figure 1, panel A.

**Table 1:** Occurrence of RSV group A and B viruses and of genotype ON1 in Kilifi, Kenya, 2012*

Month	No. RSV strains detected					RSV group A sequencing result		
	Group A	Group B	Co-infected (A+B)	Total		No. (%) ON1 strains†	Non-ON1	Not sequenced
First wave								
Jan	0	18	0	18		0	0	0
Feb	6	23	0	29		1 (20.0)	4	1
Mar	15	26	1	42		8 (53.3)	7	1
Apr	14	16	1	31		5 (41.7)	7	3
May	4	6	0	10		3 (100.0)	0	1
Jun	11	2	0	13		9 (81.8)	2	0
Jul	3	0	0	3		3 (100.0)	0	0
Aug	2	0	0	2		2 (100.0)	0	0
Non-wave period								
Sep	0	1	0	1		0	0	0
Second wave								
Oct	7	1	0	8		4 (66.7)	2	1
Nov	38	16	1	55		31 (91.2)	3	5
Dec‡	23	5	0	28		11 (84.6)	2	10
Total	123	114	3	240		77 (74.0)	27	21

*The calendar year 2012 spans parts of 2 epidemics: the 2011–12 epidemic, which occurred during October 2011–August 2012 (peak March 2012), and the 2012–13 epidemic, which occurred during October 2012–July 2013 (peak November 2012). Group B RSVs predominated during the 2011–12 epidemic (65.0%), and group A RSVs predominated during the 2012–13 epidemic (79.28%). RSV, respiratory syncytial virus. †Percentages represent the proportion of ON1 viruses among RSV A sequenced samples from each month. ‡Because of local logistics problems, samples were not collected from all the subjects that were eligible in December.

### Two Waves of RSV Genotype ON1 Infections

The ON1 genotype was first detected in Kilifi in February 2012, which was about the middle of the 2011–12 RSV epidemic. The ON1 viruses continued to be detected along with the other group A viruses through June 2012; in July and August, ON1 was the only RSV A genotype detected, marking the end of the first wave (Table 1). Overall, however, the full 2011–12 RSV epidemic was dominated by group B viruses (65.0%), which co-circulated with the group A genotypes during the epidemic (Table 1, including footnotes; Figure 1, panel A). During the first 8 months of 2012, a total of 31 ON1 infections were detected, representing 20.9% (31/148) of the overall RSV diagnoses and 60.8% (31/51) of the group A diagnoses with known ON1 status (Table 1).

The second wave of ON1 infections in Kilifi started in October 2012 at the beginning of the 2012–13 RSV epidemic, and detection continued up to the last month covered by the surveillance reported here (December 2012) (Table 1). During those 3 months, more cases of group A (75.6%, 68/90) than group B RSV were detected, and the ON1 genotype constituted the majority of the RSV A viruses (86.8%, 46/53) among those successfully sequenced (Table 1). Overall, more ON1 cases were recorded each week during the second infection wave than during the first wave (Figure 1, panel A), and the genotype appeared to predominate the other RSV A genotypes during the second wave (Table 1).

### Genetic Variability of ON1 Viruses from the Two Infection Waves

**Figure 2:**
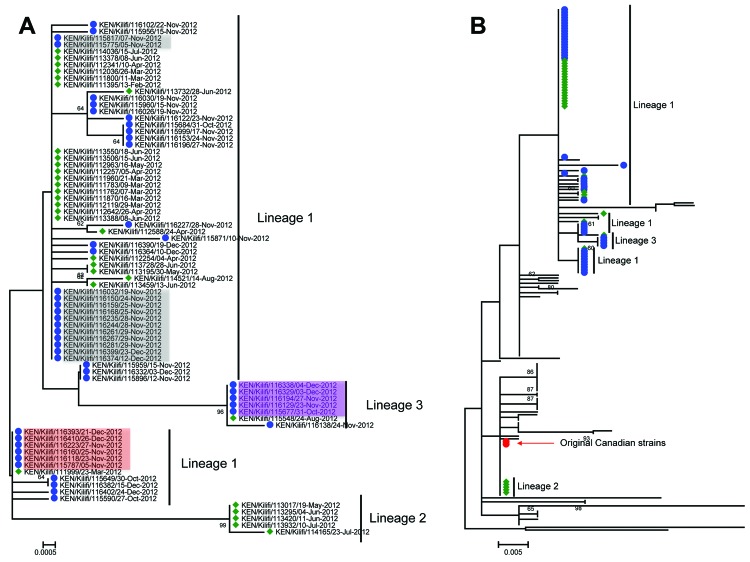
Phylogeny of respiratory syncytial virus genotype ON1 viruses detected globally and from Kilifi, Kenya. A) Maximum-likelihood, nucleotide-based phylogenetic tree showing the evolutionary relationships of the 77 Kilifi ON1 viruses across the sequenced portion (702 nt long) of the attachment (G) protein gene. The taxon nomenclature on the tree is as follows: A 3-letter code representing country of isolation/(location within country of isolation, if provided)/GenBank accession number (or identification for Kilifi sequences)/date of isolation. Kilifi viruses identified during the first infection wave are preceded by a green diamond; those identified during the second infection wave are preceded by a blue circle. Highlighted names (i.e., red, gray, and purple, indicating viruses by taxa) had sequences identical to those for viruses from the first ON1 infection wave. B) Maximum-likelihood, nucleotide-based phylogenetic tree showing the evolutionary backbone structure of 118 global ON1 viruses sequences, together with the 77 Kilifi ON1 viruses sequences across the sequenced portion (333 nt long) of the G third C-terminus region. The positions of the Kilifi viruses from the first infection wave are indicated by a green diamond, those from the second wave are indicated by a blue circle; the red circle indicates the position of the original Canadian ON1 viruses. On both trees, only bootstrap support values >60 are shown on the branches. Scale bars indicate nucleotide substitutions per site.

Of the 77 sequenced ON1 viruses, 25 unique nucleotide sequences were identified across the 702 nt–long G gene region: 8 were found only in the first infection wave, 14 were found only in the second wave, and 3 were found in both waves. Phylogenetic analysis of the G gene region of these ON1 genotype Kilifi viruses identified 3 main lineages circulating in Kilifi. The 3 lineages comprised multiple phylogenetic clusters and several singleton sequences (Figure 1, panel B; Figure 2, panel A). Lineage 1 was the first to be identified and included most (84.4%, 65/77) of the Kilifi ON1 viruses. The sequences of this lineage did not fall into a single cluster, but they were closely related and shared signature mutations (Table 2; Technical Appendix Figure 1. Lineages 2 (5 viruses) and 3 (7 viruses) were assigned from the 2 phylogenetic clusters that were prominent from the rest of the Kilifi ON1 sequences and on well-supported branches (bootstrap >90%). These lineages most likely represented 2 independent introductions of the ON1 genotype into the Kilifi community during the first infection wave. It is also possible that lineage 1 was introduced multiple times, which would explain its sequence diversity (shown by the multiple small clusters and the singleton sequences). Figure 2, panel B, shows how the 3 Kilifi lineages fit into the global picture on phylogenetic analysis of the C-terminal third region of all ON1 sequences detected throughout the world to date (see detail below).

**Table 2:** Signature codon changes in circulating global RSV group A genotype ON1 viruses*

Signature change†	First detected	No. viruses with mutation	No. viruses compared‡	Detection location(s)	Comment(s)
P88S	2012 Nov	3	7	China	Occur within the mucin-like first hypervariable region; potentially *O*-glycosylated (*4*)
L115P	2012 Aug	10	84	China, Kenya	Defines Kenyan RSV lineage 3; occurs within the mucin-like first hypervariable region; potentially *O*-glycosylated (*4*)
S128F	2012 May	5	84	Kenya	Defines Kenya RSV lineage 2
T136I	2012 Feb	54	111	Kenya	Defines Kenyan RSV lineage 1
P172A	2012 Dec	9	150	Italy	
P206Q	2011	5	162	India, Italy, Japan	
T249N	2012 Jan	5	195	Italy	
L274P	2011	131	195	India, Italy, Germany, Japan, Kenya	Predicted to be positively selected; exhibits reversible amino acid substitutions (i.e., flip-flop pattern) (*25*); position *N*-glycosylated in some strains
H290Y	2012 Feb	5	195	Italy	Predicted to be positively selected; exhibits flip-flop pattern(*25*)
G296S	2012 Dec	11	195	Italy	Adjacent codon (i.e., 295) predicted to be positively selected (*26*)
L298P	2011	133	195	India, Italy, Germany, Japan, Kenya	Duplicated epitope; concurrent substitution with L274P in many sequences
Y304H	2011	129	195	India, Italy, Germany, Japan, Kenya	
E308K	2012 Feb	13	195	Italy, South Africa, China	
L310P	2011	96	195	Italy, Kenya, Japan, India	Equivalent to L286P in non-ON1 genotypes; change previously observed in certain monoclonal antibody escape mutants (*27*)

*RSV, respiratory syncytial virus. †Only substitutions observed in >3 viruses are shown.  ‡Numbers in this column indicate the number of sequences that span the given amino acid position; the number 195 implies that all the datasets we collated, including the sequences for viruses from Kilifi, Kenya. The numbers differ because the nucleotide sequences found in GenBank for these viruses were of variable lengths.

### Persistence of ON1 Variants between Infection Waves

G nucleotide sequences for 24 of 46 ON1 viruses sequenced from the second infection wave were identical to sequences of 3 variants from the first wave (Figures 1, panel B; Figure 2, panel A), suggesting possible sustained transmission of these variants in the community through the interepidemic trough. Two of these first-wave variants were from lineage 1, and the third was from lineage 3. Phylogenetic analysis of viruses from the first and second infection waves (Figure 2, panel A) showed that lineage 1 and 3 viruses were detected during both waves, but lineage 2 was detected only during the first wave (Figure 1, panel B). These persisting first-wave variants were initially detected on February 13, March 23, and August 24, 2012, and they were still being detected in December 2012 (during the second infection wave), the last month of the surveillance reported here.

### Genetic Variability of ON1 Viruses Identified Globally

To evaluate the global genetic variability of the ON1 genotype, we combined the G gene sequences from the 77 ON1 viruses from Kenya with 118 ON1 G gene sequences in GenBank from 9 other countries; the GenBank sequences represented all ON1 sequences available as of September 8, 2013. The phylogenetic relationships of these combined ON1 sequences in the G gene are shown in Figure 2, panel B, and in Technical Appendix Figure 2 (taxon names provided). Sequences for 72 of the 77 ON1 viruses from Kenya (i.e., all lineage 1 and 3 sequences from the Kilifi data) fell into 1 branch of the global phylogeny. The bootstrap support value for the 72 sequences was low, indicating the presence of just a few unique substitutions. This branch also included 20 viruses from Japan, 2 viruses from Italy, and the 1 virus from India (Technical Appendix Figure 2). Of the 20 Japanese viruses within this branch, 12 were identical to 1 of the Kilifi variants that appeared in the first infection wave and persisted into the second wave. The ON1 Kilifi lineage that fell outside this main Kenyan branch (i.e., lineage 2) clustered with the original ON1 viruses from Canada together with ON1 viruses from Germany, Malaysia, and Italy (Figure 2, panel B). 

The reconstruction of a phylogenetic tree combining all the global ON1 sequences, from which the duplicated region had been excised, and the non-ON1 sequences detected in Kilifi in 2012 showed that the global ON1 sequences, including those from Kilifi, form a monophyletic cluster away from sequences of non-ON1 viruses. This finding reaffirmed that the Kilifi viruses with the duplication (ON1) did not arise de novo locally (data not shown).

**Figure 3:**
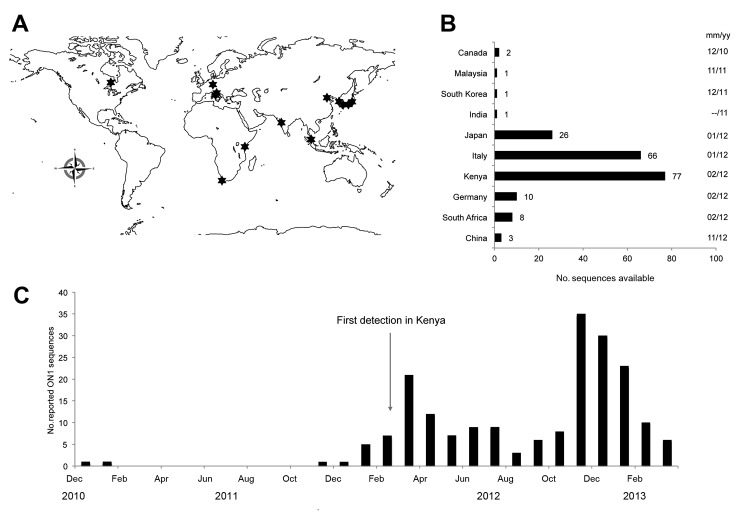
A) Geographic locations (indicated by stars) where respiratory syncytial virus genotype ON1 sequences had been detected and reported as of September 8, 2013. B) Number of ON1 sequences present in GenBank as of September 8, 2013, including sequences submitted for the viruses detected in Kilifi, Kenya, during 2012. The month and year that the first ON1 virus was reported for each country is given on the far right. C) Monthly reported detections of the ON1 viruses with sequences from the combined worldwide dataset. The month of first ON1 detection at Kilifi is indicated by an arrow. Note that the data presented in this figure have not been systematically collected to represent the geographic and temporal distribution of the ON1 genotype. Instead, the data are derived from sequence submissions to GenBank, with inherent sampling bias, that do not necessarily reflect the total number of cases from the different locations; the submissions do, however, indicate the rapid spread of the genotype.

The global geographic locations for which ON1 sequences were available in GenBank as of September 8, 2013, and the number of sequences present by country are shown in Figure 3, panels A and B. The temporal patterns for the detections of the ON1 viruses in Kilifi and in the GenBank dataset are consistent with 2 ON1 infection waves in 2012; the genotype was rare in 2011, despite first being detected in 2010 (Figure 3, panel C).

### Signature Amino Acid Substitutions

**Figure 4:**
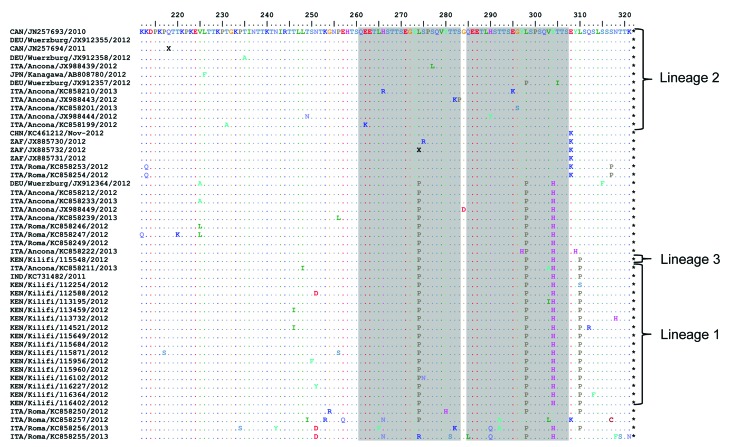
Alignment of unique deduced amino acid sequences from the combined dataset of sequences from the C-terminal third of the attachment protein of respiratory syncytial virus genotype ON1. The sequences are compared with the sequence for the earliest ON1 variant (from Ontario, Canada). The duplicated parent and the resulting regions are in gray.

Several nonsynonymous substitutions were predicted in the first and second hypervariable regions of the ON1 G protein. Signature coding mutations that were observed in >3 viruses are summarized in Table 2, and the amino acid alignment of the deduced G protein C-terminus region from the unique nucleotide sequences among the combined Kenyan ON1 and GenBank collated dataset is shown in Figure 4. Several of the changes within the second hypervariable G region had occurred on codon positions (relative to the prototype strain RSV A2) previously predicted to be positively selected: codons 225, 226, 246, 248, 249, 253, 256, 262, 265, 272, 274–276, 280, and 284–286 (*25*,*28*). Four amino acid changes (L274P, L298P, Y304H, and L310P) relative to the original Canadian viruses were shared by most of the viruses from multiple countries and defined the 2 major branches on the phylogenetic tree (Table 2; Figures 2, panel B, and 4). The first 2 of these changes, L274P and L298P, occurred concurrently in most strains and refer to the same positions within the parent and the resulting duplication region; thus, this event is considered noteworthy because the region nearby (aa 265–273) is a reported antigenic site (*29*). Furthermore, changes were observed in the region that would change the potential *N*-glycosylation profile from the original Canadian viruses. Some of the changes would cause the loss of a site (e.g., N318H in Ken/113732/28-Jun-2012 and N318F in ITA/Roma/KC858255/2013) and others would cause site gains (e.g., H266N in ITA/Roma/KC858255/2013 and ITA/Roma/KC858257/2012).

### Timing of the ON1 Genotype MRCA and Evolution Rate

**Figure 5:**
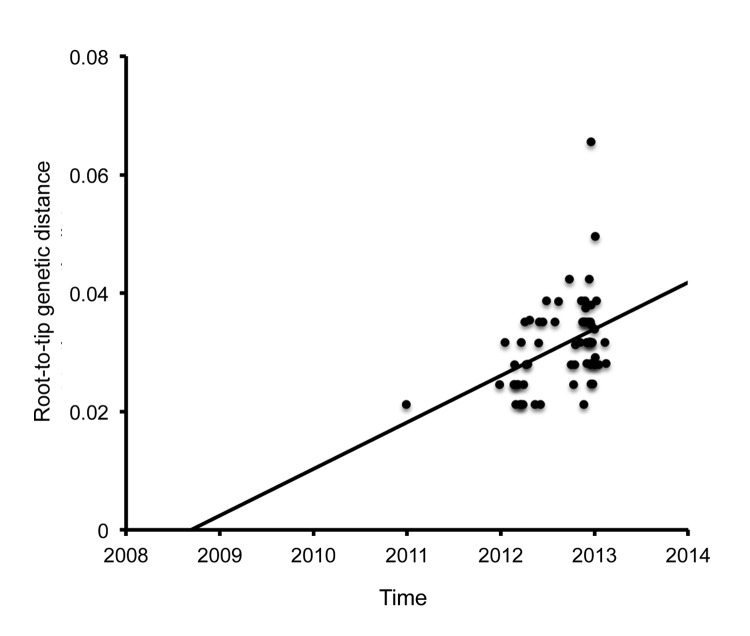
Most recent common ancestor analysis of the 65 respiratory syncytial virus genotype ON1 viruses in GenBank and the sequences for ON1 viruses detected in Kilifi, Kenya, during 2012. The analysis was done by root-to-tip regression of the genetic distances from the maximum-likelihood tree.

The MRCA analysis of the combined global ON1 sequences from the root-to-tip genetic distances on the maximum-likelihood tree showed that the ON1 genotype probably arose during the 2008–09 RSV season; the point estimate was August 2008 (Figure 5). The nucleotide substitution rate was estimated to be 7.87 × 10^−3^ substitutions per site per year in the C-terminus region. The alternative Bayesian methods showed that the global ON1 genotype viruses MRCA probably occurred in December 2009 (95% highest probability density [HPD] interval, 2004.26–2012.10), and the nucleotide substitution rate over the period was estimated to be 5.27 × 10^−3^ (95% HPD interval; 1.53 × 10^−3^ to 9.11 × 10^−3^). Although the Bayesian methods presented a wide estimate interval, both analysis methods indicate that the variant probably arose 1–2 epidemic seasons before its first detection in Ontario.

## Discussion

Our findings reinforce the observation that respiratory viruses, including RSV, can spread rapidly around the world. It has long been known that novel antigenic variants of influenza A spread rapidly over short periods and drive successive epidemics; however, the mechanism of recurrent epidemics or global endemicity for RSV is poorly understood (*30*). Observation of the emergence and rapid spread and diversification of RSV group B genotype BA (containing a 60-nt duplication) has improved our understanding of the scale of RSV spread and transmission in the world (*29*). Our findings regarding the ON1 genotype, which also possesses a large nucleotide duplication, closely relate to findings regarding the BA genotype and confirm that novel RSV strains do spread rapidly and widely.

Of particular interest is the observation that within a few years of the generation of these new genotypes, variants have evolved with accumulated signature coding changes in regions of the G protein targeted by the neutralizing antibody response. For example, a previously identified putative epitope around codon 274 was duplicated in the ON1 genotype, and some of the emerging variants have shown amino acid changes in both copies of this epitope (Figure 4). This suggests that such ON1 variants were selected by the change on this particular epitope. Furthermore, in many ON1 variants, additional coding changes are observed at codons previously predicted to be positively selected (*25*,*26*,*28*), at potential *N*-glycosylation codons (*4*), and at positions previously reported in escape mutants from certain monoclonal antibodies (e.g., L310P, which in viruses without the duplication will be equivalent to L286P) (*27*). The P286L change has been associated with abrogation of reaction of peptides to convalescent-phase human serum (*31*). Thus it may be deduced that the amino acid changes already observed in the ON1 variants have led to profound differences in its antigenic profile.

The presence of the multiple ON1 genotype lineages in Kilifi, some occurring as multiple phylogenetic clusters or several distinct sequences, suggests that there have been multiple introductions of the genotype into the region. Some of these ON1 viruses have partial G gene sequences identical to the few ON1 genotype GenBank sequences available from around the world, indicating that ON1 variants that arose soon after its emergence are also quickly spreading worldwide. We also detected identical ON1 G gene sequences for viruses from the 2 RSV infection waves in Kilifi, which could reflect continued local transmission of the first-wave viruses or further new introductions of identical viruses of the genotype into the community during the second wave.

MRCA analysis determined that ON1 first emerged in 2008 or 2009. This would suggest that the variant was circulating undetected before December 2010, and its first location of occurrence may remain unknown. This estimate of the time between first occurrence and detection is shorter than the ≈12 years proposed by Tsukagoshi et al. (*13*). Overall, by both the maximum likelihood and Bayesian methods, we estimated that this variant had a higher rate of evolution in its C-terminus region (point estimates of >5.0 × 10^−3^ nucleotide substitutions per site per year) than previously predicted for RSV A (3.382 × 10^−3^ [95% HPD interval 1.911 × 10^−3^ to 4.954 × 10^−3^]) (*32*). This finding suggested an accelerated evolution rate in this variant early in its lifetime; however, our estimates have wide intervals, and it should be noted that viruses with G gene nucleotide sequences nearly identical to those of the original Canadian ON1 viruses were co-circulating with the diversified viruses at least up to late in 2012.

Our RSV ON1 genotype analyses were limited to the G ectodomain region (≈700 nt), and analyses for the global data were limited to the G C-terminus region (≈330 nt). Whole-genome comparison of 2 ON1 sequences currently available in GenBank (accession nos. JX627336/2011 [*12*] and KC731482/2011 [*11*]) showed up to 57 nt differences, a substantial proportion of which occurred beyond the G protein gene region (data not shown). Thus, to better understand the local and global molecular epidemiology and phylogeography of the ON1 genotype, whole-genome sequences must be compared. To that end, we are currently conducting whole-genome sequencing of the ON1 viruses from Kilifi.

The phylodynamics of these emergent RSV genotypes with large nucleotide duplications in the G gene (i.e., the BA and ON1) enable parallels to be drawn with pandemic influenza viruses arising from antigenic shift. The cause of the apparent enhanced biologic fitness of the BA and ON1 genotypes (whether virologic or immunologic) is not well understood. Since around 2005, the BA genotype has dominated all other group B genotypes. We cannot tell if the ON1 genotype will also eventually dominate other group A genotypes. However, during the preparation of this report, the detection of RSV genotype ON1 was reported in 3 more countries: Thailand (*33*), Latvia (*34*), and Cyprus (*35*). Thus the prevalence and geographic distribution of ON1 is rapidly changing. The monitoring of this change will lead to a better understanding of the factors underlying the successful emergence of variant genotypes and help inform future methods for the control of RSV.

Technical Appendix Signature mutations that distinguished the 3 respiratory syncytial virus ON1 lineages identified at Kilifi, Kenya, taxon names, and mean genetic distances between the 3 lineages.

## References

[1] Collins PL, Melero JA. Progress in understanding and controlling respiratory syncytial virus: still crazy after all these years. Virus Res. 2011;162:80–99. 10.1016/j.virusres.2011.09.020 21963675 PMC3221877

[2] Cane PA, Matthews DA, Pringle CR. Analysis of respiratory syncytial virus strain variation in successive epidemics in one city. J Clin Microbiol. 1994;32:1–4 . 8126162 10.1128/jcm.32.1.1-4.1994 PMC262959

[3] Cane PA, Pringle CR. Evolution of subgroup A respiratory syncytial virus: evidence for progressive accumulation of amino acid changes in the attachment protein. J Virol. 1995;69:2918–25 . 7707517 10.1128/jvi.69.5.2918-2925.1995 PMC188990

[4] Melero JA, Garcia-Barreno B, Martinez I, Pringle CR, Cane PA. Antigenic structure, evolution and immunobiology of human respiratory syncytial virus attachment (G) protein. J Gen Virol. 1997;78:2411–8 . 9349459 10.1099/0022-1317-78-10-2411

[5] Smith GJ, Vijaykrishna D, Bahl J, Lycett SJ, Worobey M, Pybus OG, Origins and evolutionary genomics of the 2009 swine-origin H1N1 influenza A epidemic. Nature. 2009;459:1122–5. 10.1038/nature08182 19516283

[6] Trento A, Galiano M, Videla C, Carballal G, Garcia-Barreno B, Melero JA, Major changes in the G protein of human respiratory syncytial virus isolates introduced by a duplication of 60 nucleotides. J Gen Virol. 2003;84:3115–20. 10.1099/vir.0.19357-0 14573817

[7] Trento A, Casas I, Calderon A, Garcia-Garcia ML, Calvo C, Perez-Brena P, Ten years of global evolution of the human respiratory syncytial virus BA genotype with a 60-nucleotide duplication in the G protein gene. J Virol. 2010;84:7500–12. 10.1128/JVI.00345-10 20504933 PMC2897623

[8] Trento A, Viegas M, Galiano M, Videla C, Carballal G, Mistchenko AS, Natural history of human respiratory syncytial virus inferred from phylogenetic analysis of the attachment (G) glycoprotein with a 60-nucleotide duplication. J Virol. 2006;80:975–84. 10.1128/JVI.80.2.975-984.2006 16378999 PMC1346866

[9] Eshaghi A, Duvvuri VR, Lai R, Nadarajah JT, Li A, Patel SN, Genetic variability of human respiratory syncytial virus A strains circulating in Ontario: a novel genotype with a 72 nucleotide G gene duplication. PLoS ONE. 2012;7:e32807. 10.1371/journal.pone.0032807 22470426 PMC3314658

[10] Khor CS, Sam IC, Hooi PS, Chan YF. Displacement of predominant respiratory syncytial virus genotypes in Malaysia between 1989 and 2011. Infect Genet Evol. 2013;14:357–60. 10.1016/j.meegid.2012.12.017 23305888

[11] Choudhary ML, Wadhwa BS, Jadhav SM, Chadha MS. Complete genome sequences of two human respiratory syncytial virus genotype A strains from India, RSV-A/NIV1114046/11 and RSV-A/NIV1114073/11. Genome Announc. 2013;1:e00165–13. 10.1128/genomeA.00165-13 PMC3735064 23887906

[12] Lee WJ, Kim YJ, Kim DW, Lee HS, Lee HY, Kim K. Complete genome sequence of human respiratory syncytial virus genotype A with a 72-nucleotide duplication in the attachment protein G gene. J Virol. 2012;86:13810–1. 10.1128/JVI.02571-12 23166231 PMC3503102

[13] Tsukagoshi H, Yokoi H, Kobayashi M, Kushibuchi I, Okamoto-Nakagawa R, Yoshida A, Genetic analysis of attachment glycoprotein (G) gene in new genotype ON1 of human respiratory syncytial virus detected in Japan. Microbiol Immunol. 2013;57:655–9 . 23750702 10.1111/1348-0421.12075

[14] Prifert C, Streng A, Krempl CD, Liese J, Weissbrich B. Novel respiratory syncytial virus A genotype, Germany, 2011–2012. Emerg Infect Dis. 2013;19:1029–30. 10.3201/eid1906.121582 23735287 PMC3713827

[15] Valley-Omar Z, Muloiwa R, Hu NC, Eley B, Hsiao NY. Novel respiratory syncytial virus subtype ON1 among children, Cape Town, South Africa, 2012. Emerg Infect Dis. 2013;19:668–70. 10.3201/eid1904.121465 23750827 PMC3647422

[16] Agoti CN, Gitahi CW, Medley GF, Cane PA, Nokes DJ. Identification of group B respiratory syncytial viruses that lack the 60-nucleotide duplication after six consecutive epidemics of total BA dominance at coastal Kenya. Influenza Other Respir Viruses. 2013;7:1008–12. 10.1111/irv.12131 PMC3963446 23782406

[17] Hammitt LL, Kazungu S, Morpeth SC, Gibson DG, Mvera B, Brent AJ, A preliminary study of pneumonia etiology among hospitalized children in Kenya. Clin Infect Dis. 2012;54(Suppl 2):S190–9. 10.1093/cid/cir1071 22403235 PMC3297554

[18] Nokes DJ, Ngama M, Bett A, Abwao J, Munywoki P, English M, Incidence and severity of respiratory syncytial virus pneumonia in rural Kenyan children identified through hospital surveillance. Clin Infect Dis. 2009;49:1341–9. 10.1086/606055 19788358 PMC2762474

[19] Munywoki PK, Hamid F, Mutunga M, Welch S, Cane P, Nokes DJ. Improved detection of respiratory viruses in pediatric outpatients with acute respiratory illness by real-time PCR using nasopharyngeal flocked swabs. J Clin Microbiol. 2011;49:3365–7. 10.1128/JCM.02231-10 21775539 PMC3165583

[20] Scott PD, Ochola R, Ngama M, Okiro EA, Nokes DJ, Medley GF, Molecular epidemiology of respiratory syncytial virus in Kilifi District, Kenya. J Med Virol. 2004 t;74:344–54. 10.1002/jmv.20183 15332285

[21] Agoti CN, Mwihuri AG, Sande CJ, Onyango CO, Medley GF, Cane PA, Genetic relatedness of infecting and reinfecting respiratory syncytial virus strains identified in a birth cohort from rural Kenya. J Infect Dis. 2012;206:1532–41. 10.1093/infdis/jis570 22966119 PMC3475639

[22] Katoh K, Toh H. Recent developments in the MAFFT multiple sequence alignment program. Brief Bioinform. 2008;9:286–98. 10.1093/bib/bbn013 18372315

[23] Tamura K, Peterson D, Peterson N, Stecher G, Nei M, Kumar S. MEGA5: Molecular Evolutionary Genetics Analysis using maximum likelihood, evolutionary distance, and maximum parsimony methods. Mol Biol Evol. 2011;28:2731–9. 10.1093/molbev/msr121 21546353 PMC3203626

[24] Drummond AJ, Rambaut A. BEAST: Bayesian evolutionary analysis by sampling trees. BMC Evol Biol. 2007;7:214. 10.1186/1471-2148-7-214 17996036 PMC2247476

[25] Botosso VF, Zanotto PM, Ueda M, Arruda E, Gilio AE, Vieira SE, Positive selection results in frequent reversible amino acid replacements in the G protein gene of human respiratory syncytial virus. PLoS Pathog. 2009;5:e1000254. 10.1371/journal.ppat.1000254 19119418 PMC2603285

[26] Woelk CH, Holmes EC. Variable immune-driven natural selection in the attachment (G) glycoprotein of respiratory syncytial virus (RSV). J Mol Evol. 2001;52:182–92 . 11231898 10.1007/s002390010147

[27] Palomo C, Cane PA, Melero JA. Evaluation of the antibody specificities of human convalescent-phase sera against the attachment (G) protein of human respiratory syncytial virus: influence of strain variation and carbohydrate side chains. J Med Virol. 2000;60:468–74. 10.1002/(SICI)1096-9071(200004)60:4<468::AID-JMV16>3.0.CO;2-E 10686032

[28] Zlateva KT, Lemey P, Vandamme AM, Van Ranst M. Molecular evolution and circulation patterns of human respiratory syncytial virus subgroup A: positively selected sites in the attachment G glycoprotein. J Virol. 2004;78:4675–83. 10.1128/JVI.78.9.4675-4683.2004 15078950 PMC387670

[29] Cane P. Molecular epidemiology and evolution of RSV. In: Cane P, editor. Respiratory syncytial virus. Amsterdam: Elsevier; 2007. p. 89–113.

[30] Medley GF, Nokes DJ. Does viral diversity matter? Science. 2009;325:274–5. 10.1126/science.1177475 19608903

[31] Cane PA. Analysis of linear epitopes recognised by the primary human antibody response to a variable region of the attachment (G) protein of respiratory syncytial virus. J Med Virol. 1997;51:297–304. 10.1002/(SICI)1096-9071(199704)51:4<297::AID-JMV7>3.0.CO;2-0 9093944

[32] van Niekerk S, Venter M. Replacement of previously circulating respiratory syncytial virus subtype B strains with the BA genotype in South Africa. J Virol. 2011;85:8789–97 . 10.1128/JVI.02623-10 21715483 PMC3165815

[33] Auksornkitti V, Kamprasert N, Thongkomplew S, Suwannakarn K, Theamboonlers A, Samransamruajkij R, Molecular characterization of human respiratory syncytial virus, 2010–2011: identification of genotype ON1 and a new subgroup B genotype in Thailand. Arch Virol. 2014;159:499–507. 10.1007/s00705-013-1773-9 24068580

[34] Balmaks R, Ribakova I, Gardovska D, Kazaks A. Molecular epidemiology of human respiratory syncytial virus over three consecutive seasons in Latvia. J Med Virol. 2013. Epub 2013 Dec 2. 10.1002/jmv.23855 24301088

[35] Panayiotou C, Richter J, Koliou M, Kalogirou N, Georgiou E, Christodoulou C. Epidemiology of respiratory syncytial virus in children in Cyprus during three consecutive winter seasons (2010–2013): age distribution, seasonality and association between prevalent genotypes and disease severity. Epidemiol Infect. 2014;24:1–6. 10.1017/S0950268814000028 24476750 PMC9151279

