# Supplementary material for: Rapid Spread and Diversification of Respiratory Syncytial Virus Genotype ON1, Kenya
Source: Emerg Infect Dis. 2014 Jun;20(6):950–9. doi: 10.3201/eid2006.131438 (PMC4036793; doi:10.3201/eid2006.131438)
Supplement: Technical Appendix — Signature mutations that distinguished the 3 respiratory syncytial virus ON1 lineages identified at Kilifi, Kenya, taxon names, and mean genetic distances between the 3 lineages. [file 13-1438-Techapp-s1.pdf]

Article DOI: http://dx.doi.org/10.3201/eid2006.131438

Rapid Spread and Diversification of the New Respiratory Syncytial ON1 Genotype Virus, Kenya

Technical Appendix

(A) Signature coding changes defining the Kenyan lineages

AA position	115	128	136	274	298	304	310
Canadian S.	L	S	T	L	L	Y	L
Lineage 1	L	S	T/I	P	P	H	P/S
Lineage 2	L	F	T	L	L	Y	L
Lineage 3	P	S	T	P	P	H	P

(B) Mean genetic distances between the defined lineages

	Lineage 1	Lineage 2	Lineage 3
Lineage 1	-		
Lineage 2	0.011	-	
Lineage 3	0.008	0.015	-

Technical Appendix Figure 1. A) Summary of signature mutations that distinguished the defined 3 respiratory syncytial virus ON1 lineages observed at Kilifi, Kenya. B) The mean genetic distances between the 3 lineages identified at Kilifi.

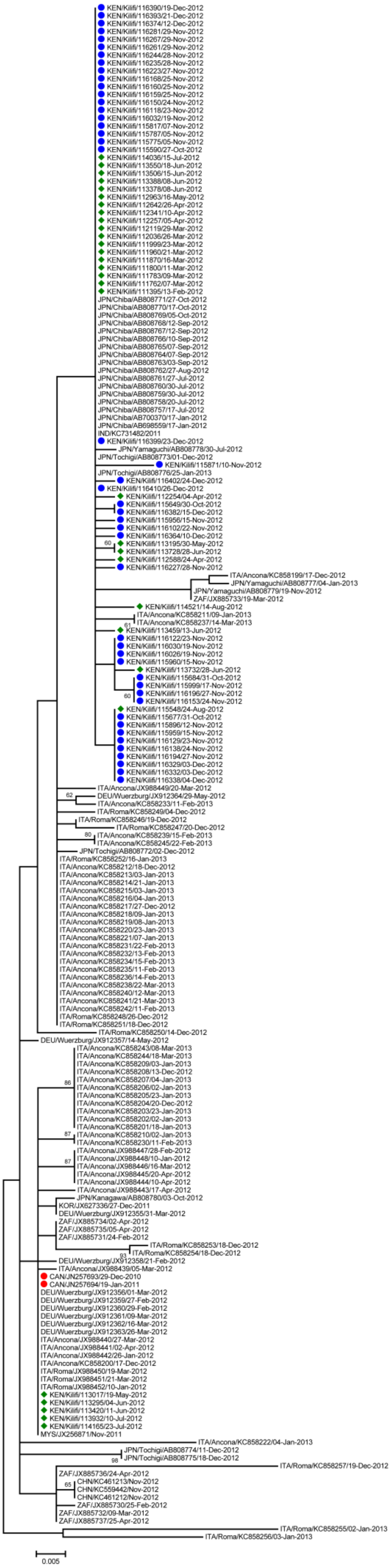

Technical Appendix Figure 2. Same as Figure 2B but showing the taxon names. The taxon nomenclature on the tree is as follows: A three letter code representing country of isolation/(location within country of isolation if provided)/ GenBank accession number (or ID for Kilifi sequences)/ Date of isolation. Only bootstrap support values above 60 are shown on the branches.
